# Systematic optimisation of crude buccal swab lysate protocols for use with the ForenSeq™ DNA Signature Prep Kit

**DOI:** 10.1007/s00414-024-03405-x

**Published:** 2025-01-13

**Authors:** Donna-Lee Pamela Martin, Laura Jane Heathfield

**Affiliations:** Division of Forensic Medicine and Toxicology, Department of Pathology, Faculty of Health Science, University of Cape Town, Cape Town, South Africa

**Keywords:** Promega SwabSolution, QIAGEN STR GO! Lysis buffer, ForenSeq^T^^M^ DNA Signature Prep kit, Direct PCR, Population study, Optimisation

## Abstract

The ForenSeq™ DNA Signature Prep kit has not been thoroughly tested with crude buccal swab lysates in large-scale population studies using massively parallel sequencing (MPS). Commonly used lysis buffers for swabs intending to undergo direct polymerase chain reaction (PCR) are SwabSolution™ and STR GO! Lysis Buffers, and these have been successfully used to generate population data using capillary electrophoresis (CE) systems. In this study, we investigated the performance and optimisation of SwabSolution™ and STR GO! lysates with the ForenSeq™ DNA Signature Prep workflow and addressed the challenge of failed MPS profiles in initial trials. To mitigate PCR inhibition in SwabSolution™ lysates, three optimisation methods were evaluated: dilution of lysates, addition of 5X AmpSolution^®^ reagent, and purification with magnetic beads. For STR GO! lysates, we explored spin-column purification using the QIAamp^®^ DNA Investigator kit, magnetic bead purification, and a pH adjustment with 1 M hydrochloric acid. Our findings indicated that the addition of 5X AmpSolution^®^ was effective for overcoming PCR inhibition in SwabSolution™ lysates, thereby maintaining a direct PCR approach. Spin-column purification, however, is recommended for STR GO! lysates to minimise MPS profile failure rates. These improvements enhance first-time success rates of crude swab lysates, and reduce the need for repeat sampling and re-sequencing, making the workflow more suitable for large-scale population studies in forensic laboratories.

**Graphical Abstract:**

*Overview of the adaptations made to ensure a high-first time success rate with crude buccal swab lysates using the ForenSeq*™* DNA Signature Prep kit workflow*

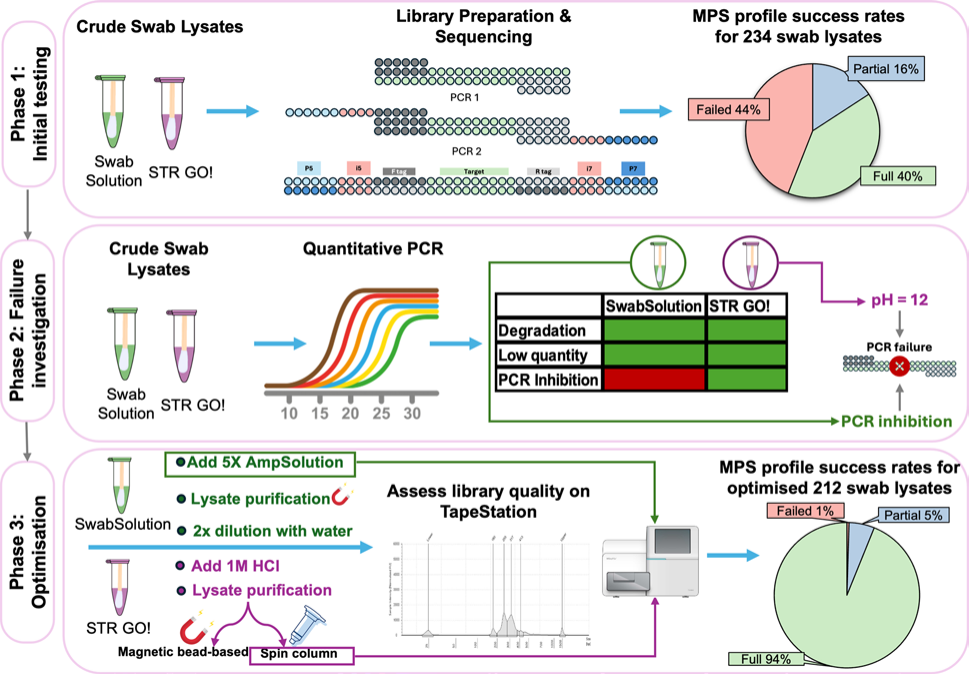

## Introduction

The requirement for cost-efficient, quick and reliable methods for generating DNA profiles, particularly for reference samples and allele frequency databases, resulted in the emergence of direct PCR methods for capillary electrophoresis (CE)-based DNA profiling [1]. Direct PCR eliminates the need for DNA extraction and DNA quantification, thereby reducing the hands-on time and costs associated with sample preparation prior to PCR and DNA profiling [2]. With regards to CE-based DNA profiling, previous studies have addressed, reviewed and evaluated the use and performance of direct PCR methods as applied to both reference and casework samples in forensic laboratories [3, 4]. These include blood, buccal swabs, soft tissue, hair, nails and trace DNA from various substrates [3–9]. For the purposes of generating DNA profiles for establishing population databases, good quality samples such as blood and buccal swabs are routinely processed with direct PCR methods to generate DNA profiles [5, 8].

The general method for processing buccal swabs with a direct PCR approach starts with a short lysis step, wherein the cells contained on a swab are separated from its substrate and lysed in a buffer at a specific temperature [9, 10]. The resulting sample is known as a crude buccal swab lysate that can be added directly to a PCR reaction for amplification and subsequent profiling (CE or massively parallel sequencing (MPS)). However, the risk associated with direct PCR methods is that, in the absence of purification, quantification and quality control steps, PCR inhibitors, such as impurities, or even sup-optimal buffer chemistry may cause interference with efficient PCR amplification [1].

Direct PCR methods have demonstrated success rates comparable to traditional approaches that involve DNA extraction and quantification prior to conventional DNA profiling using CE. However, some studies report lower first-pass success rates for samples processed using direct PCR workflows [5, 11, 12]. The polymerases and PCR buffers in certain commercial short tandem repeat (STR) profiling kits are formulated to tolerate some degree of inhibition and have built-in buffering capacity to maintain polymerase activity [13]. In contrast, forensic kits designed for MPS are significantly more sensitive and may be compromised by PCR inhibitors and suboptimal polymerase conditions [14]. Without purification, quantification, and library quality assessment, the likelihood of reduced success rates with sensitive MPS kit chemistries increases [1]. MPS kits may be more sensitive because they target and amplify thousands of DNA fragments simultaneously to efficiently amplify challenging samples. This sensitivity, however, may increase their susceptibility to PCR inhibitors. CE kits, in contrast, have undergone decades of optimisation for inhibitor tolerance and are supported by tailored chemistries, for example, the QIAGEN STR GO! lysis buffer, developed to be compatible with the QIAGEN Investigator® 24Plex GO! kit. Forensic MPS kits, still in their developmental stages, lack such integration and compatibility. The trade-off between sensitivity and tolerance to inhibitors in MPS workflows may require further refinement to match the balance achieved in CE systems.

The ForenSeq™ DNA Signature Prep kit (Verogen, San Diego, CA, USA) has been developmentally validated for direct PCR methods, although direct PCR methods are not used as often as conventional DNA extraction methods in large-scale MPS studies [16]. To date, no MPS-based population studies have used crude buccal swab lysates with the ForenSeq™ DNA Signature Prep kit, and it is therefore currently unclear how crude buccal swab lysates perform with this MPS workflow for large-scale studies. Crude buccal swab lysates generated using the SwabSolution™ kit (Promega Corporation, WI, USA) and the STR GO! Lysis buffer (QIAGEN, Hilden, Germany) are commonly used as reference samples, and have been used to successfully carry out population studies with CE systems using commercial STR profiling kits [17, 18]. It was assumed that these same samples would perform similarly using the ForenSeq™ DNA Signature Prep kit (Verogen), as the manufacturer’s had tested crude buccal swab lysates and provided input recommendations in the manufacturer’s protocol. The ForenSeq™ DNA Signature Prep kit manufacturer’s protocol states that the SwabSolution™ kit (Promega) has been validated for use with the ForenSeq™ DNA Signature Prep kit and recommends adding 3 µL water and 2 µL of the crude buccal swab lysate directly to the PCR 1 reaction, without prior assessment or quantification of the lysate, and without library quality assessment [19]. A population study was thus initially attempted using the crude buccal swab lysates generated with the SwabSolution™ kit (Promega) (hereon referred to as “SwabSolution™ lysates”) and with the STR GO! Lysis Buffer (QIAGEN) (hereon referred to as “STR GO! lysates”) with the ForenSeq™ DNA Signature Prep kit (Verogen) which resulted in failed MPS profiles. The aim of this study was thus to investigate and optimise first-time success rates of crude buccal swab lysates generated using the SwabSolution™ kit (Promega) and the STR GO! Lysis buffer (QIAGEN) that have been processed with the ForenSeq™ DNA Signature Prep kit (Verogen) in preparation for use in large-scale MPS studies.

## Methods

### Phase 1: Initial processing of crude buccal swab lysates with the ForenSeq™ DNA Signature prep kit

In an ethically approved, ongoing study at the University of Cape Town (HREC: 342/2016), cotton buccal swabs were collected from both living and deceased individuals, as detailed by Heathfield et al., 2024. A total of 500 swabs were processed using the SwabSolution™ Kit (Promega), and another 500 with the STR GO! Lysis Buffer (QIAGEN), according to the respective manufacturer’s guidelines [17, 18]. Full conventional DNA profiles were generated for these samples as reported by Heathfield et al., 2024, using CE methods [18]. Crude buccal swab lysates were processed (*n* = 234) with the ForenSeq™ DNA Signature Prep kit (Primer mix A and/or B), as part of an MPS population study for the South African population (Martin et al.., manuscript under review). Libraries were purified, normalised, denatured and loaded as per the manufacturer’s protocol, except that a loading volume of 12 µL of pooled, normalised library was used, as determined through prior internal laboratory optimisation experiments [18]. Approximately 600 µL of denatured Human Sequencing Control (HSC) and pooled, normalised library were loaded onto a MiSeq FGx™ reagent cartridge and sequenced on the MiSeq FGx™ instrument (Verogen) for 398 cycles in Forensic Genomics mode according to the manufacturer’s protocol [21].

#### Data analysis

Primary data analysis was performed on the ForenSeq™ UAS [22]. Alleles were automatically called based on their read counts and whether the read count met the default analytical and interpretation thresholds of 1.5% and 4.5% respectively. Where allele calls for potentially heterozygous genotypes were above the analytical threshold, but below the interpretation threshold, manual editing was performed to include the second allele back into the genotype. The modified sample genotype reports were used to determine call rates. Call rate was calculated as the number of successfully called genotypes/haplotypes at each marker, divided by the total number of markers, and converted to a percentage. Successfully called genotypes excluded instances of allele dropout at a locus (i.e., allele dropout was considered as an unsuccessful genotype).

### Phase 2: investigation of crude buccal swab lysate failure

#### Real-time PCR quantification

SwabSolution™ (Promega) and STR GO! (QIAGEN) lysates that produced failed or partial MPS profiles using the ForenSeq™ DNA Signature Prep kit (Verogen), were processed with the Quantifiler^®^ Trio kit (Applied Biosystems, Foster City, USA), using half-volumes [23]. It was initially hypothesised that PCR inhibition played a role in suboptimal lysate performance, therefore initial assessment of lysates included an assessment of PCR inhibition using real time PCR (qPCR). Lysates that were flagged for high internal positive control (IPC) cycle threshold (C_T_) (i.e., IPC C_T_ > 31) values by the HID real-time PCR software were diluted two-fold with molecular biology grade water prior to re-quantification with the Quantifiler^®^ Trio kit (Applied Biosystems) to confirm inhibition. Concentration and degradation status of lysates were also assessed.

#### pH assessment of STR GO! Buccal swab lysates

Whilst helpful for the buccal swabs prepared in SwabSolution™ (Promega), the qPCR results provided little to no insight into MPS profile failure with STR GO! lysates. It was therefore hypothesised that suboptimal performance of these lysates could be due to the incompatibility of lysis buffers with the ForenSeq™ DNA Signature Prep kits’ PCR 1 components. Further investigation into this incompatibility involved personal communication with the manufacturers to understand reasons behind potential incompatibility of buffers, and it was noted that the high pH of the STR GO! Lysis buffer (QIAGEN) could not be negated due to the limited buffering capacity of ForenSeq™ DNA Signature Prep kit pre-PCR reagents (Personal Communication, QIAGEN, Hilden, Germany).

### Phase 3: method optimisation

#### SwabSolution™ crude buccal swab lysates

As PCR inhibition was confirmed in SwabSolution™ lysates in phase 2, a subset of 10 SwabSolution™ lysates that had failed MPS profiles were subjected to three different optimisation methods prior to library preparation with the ForenSeq™ DNA Signature Prep kit (Verogen):

- A two-fold dilution of crude buccal swab lysates with nuclease-free water prior to library preparation was performed [19].
- The addition of 3 µL 5X AmpSolution^®^ reagent (Promega) to 2 µL of the crude buccal swab lysate (instead of 3 µL nuclease-free water).
- Purification of 100 µL of lysate using the Mag-Bind^®^ Blood DNA HV kit (Omega Bio-tek, Norcross, GA, USA) eluted into 30 µL of TE buffer [24].

#### Lysate preparation methods for STR GO! Buffer lysates

To overcome the high pH of the STR GO! lysates, 10 STR GO! crude buccal swab lysates that had failed or suboptimal MPS call rates were subjected to three optimisations.

- Purification of 100 µL of lysate using the QIAamp^®^ DNA Investigator kit, eluted into 30 µL of ATE buffer (QIAGEN, Hilden, Germany) [25].
- Purification of 100 µL of lysate using the Mag-Bind^®^ Blood DNA HV kit (Omega Bio-tek) eluted into 30 µL of TE buffer [24].
- Addition of 3 µL 1 M hydrochloric acid (HCl) (Sigma-Aldrich) to 2 µL of the crude buccal swab lysate (instead of 3 µL nuclease-free water) to reduce the pH without further dilution of the lysate.

#### Quantification of extracted DNA

Where lysates were purified, DNA was quantified with the Quantifiler^®^ Trio Kit (Applied Biosystems) and diluted to 0.2 ng/µL with nuclease-free water.

### Library preparation

Extracted DNA and crude buccal swab lysates prepared in phase 3 were processed using the ForenSeq™ DNA Signature Prep kit (Verogen), as stipulated in the manufacturer’s protocol, with modifications as presented in phase 3 above [19]. All libraries were prepared alongside a positive control using 5 µL of the 2800M control DNA (Promega), which was prepared by dilution to 0.2 ng/µL with nuclease-free water, along with a negative control consisting of 5 µL nuclease-free water. The amplification and tagging of targets, enrichment of targets and library purification was performed according to the manufacturer’s guidelines. Purified libraries were stored in a −20 ℃ freezer until further use.

### Assessment of library quality and quantity of crude buccal swab lysates

The ‘safe stopping point’ (as per the manufacturer’s protocol) was used to assess the quality and quantity of purified libraries [19]. The purified libraries of the positive control, the lysates subjected to the different library preparation methods as well as previously processed lysates that had initially resulted in failed or partial profiles were processed using the High Sensitivity D1000 Screen Tape Assay with the 2200 TapeStation (Agilent Technologies) following the manufacturer’s protocol [26]. The average library sizes were recorded from the TapeStation Controller Software. Library concentration was assessed using the Qubit 2.0 Fluorometer with the Qubit™ 1x dsDNA High Sensitivity Assay (Thermo Fisher Scientific, Waltham, MA, USA) [27].

### Statistical analysis for assessment of optimisation methods

To evaluate whether the lysate preparation method used influenced library quantity and average library size, two-sided Friedman’s tests were performed after a Shapiro-Wilk test revealed that the assumption of normality was not met. Conover’s test was used for post-hoc comparison. This was used to test for significant differences in library concentration and library sizes between the different lysate preparation methods. A significance level of 0.05 was used. To determine the most optimised protocol for STR GO! purified lysates, the library concentration and average library sizes of the purified libraries were compared to the 2800M control purified library metrics (library concentration and size) using a one-sample Wilcoxon test, as both used the same input concentration of 0.2 ng/µL. To evaluate statistical differences in the original method, and the optimised method selected for large-scale sequencing, a Mann-Whitney U test was performed. A significance level of 0.05 was used for all tests. All statistical analyses were conducted using the SciPy. Stats package in Python version 3.7 [28].

### Library normalisation and sequencing post optimisation

Following statistical assessment of the lysate preparation method best suited to each type of lysate (SwabSolution™ and STR GO! lysates), the selected purified libraries were normalised and sequenced on the MiSeq FGx™ sequencer in batches of 32 according to the manufacturer’s protocol. A total of 212 samples were sequenced (including previously failed crude buccal swab lysates) using the optimised protocols and call rates compared to the previously failed crude buccal swab lysates.

## Results

### Phase 1: initial assessment of failure rates

#### Quality metrics

The quality metrics for the seven experimental runs that included lysates processed in phase 1 are shown in Table 1. All quality metrics were within the recommended ranges reported by the manufacturer, except for three runs, where cluster density was low [21].

**Table 1:** Quality metrics obtained for sequencing runs performed with the ForenSeq™ DNA signature Prep kit on the MiSeq FGx™ sequencer. Bold text indicates values that were flagged for low cluster density. The manufacturer’s recommended ranges are shown in square brackets [21]

Experiment Number	Cluster Density (K/mm^2^) [400–1650 K/mm^2^]	Clusters Passing Filter (%) [≥ 80%]	Phasing (%) [≤ 0.25%]	Pre-phasing (%) [≤ 0.15%]
**Run 1**	777	93.25	0.132	0.093
**Run 2**	1069	91.00	0.112	0.095
**Run 3**	**681**	92.63	0.158	0.113
**Run 4**	**456**	96.35	0.219	0.098
**Run 5**	1000	88.95	0.156	0.115
**Run 6**	1187	87.87	0.143	0.114
**Run 7**	**520**	91.07	0.205	0.094

#### Call rates

Preliminary assessment of call rates of crude buccal swab lysates processed with the ForenSeq™ DNA Signature Prep kit revealed that a substantial portion of crude buccal swab lysates resulted in failed or partial profiles. The failed profiles accounted for 44% (*n* = 103) of the crude buccal swab lysates processed, 16% (*n* = 37) were partial profiles, and 40% (*n* = 94) were full profiles. SwabSolution™ lysates accounted for 61% (*n* = 63) of failed samples, while STR GO! lysates made up 39% (*n* = 40).

### Phase 2: investigation of lysate failure

#### SwabSolution™ crude buccal swab lysates

The subset of lysates (*n* = 103) that resulted in failed MPS profiles were categorised into lysate type; SwabSolution™ lysates and STR GO! lysates. Real-time qPCR results showed that 93.65% (*n* = 59/63) of SwabSolution™ lysates had IPC C_T_ values above 31 and were thus likely to be affected by PCR inhibition. The degradation index (DI) values for SwabSolution™ lysates indicated that the lysates were unlikely degraded, as most samples had DI values below four. Degradation was thus ruled out as a possible reason for failure. The DI categories proposed by Vernarecci et al., 2015 were used in this study [29]. Additionally, where samples were not affected by inhibition, concentrations were within adequate ranges for sequencing. It should be noted that many lysates that were flagged for inhibition had unknown DI and concentration values, therefore concentration and DI values could not be properly assessed for those samples. In these cases, the reason for failure with MPS methods was taken as inhibition. Dilution of SwabSolution™ crude buccal swab lysates that were identified as potentially inhibited showed an improvement in the IPC C_T_ value, where 87.30% (*n* = 55/63) of lysates had new IPC C_T_ values < 30, supporting the hypothesis that these samples were initially inhibited.

#### STR GO! Crude buccal swab lysates

The real-time PCR results for STR GO! (QIAGEN) crude buccal swab lysates showed that 100% of lysates had an IPC C_T_ value below 31 and were thus *unlikely* to be inhibited. The DI values indicated little to no degradation. Concentrations were above 0.2 ng/µL in all samples. The failure of lysates with MPS could thus not be attributed to low input concentration, degradation or inhibition. Further investigation into reasons for failure involved personal communication with the manufacturers, during which it was established that the pH of the STR GO! Lysis buffer (QIAGEN) was too high to be compatible with the ForenSeq™ DNA Signature Prep kit PCR 1 buffers (Personal communication, QIAGEN, Hilden, Germany). This information was pivotal to establish methods to improve compatibility between the STR GO! Lysis buffer (QIAGEN) and the ForenSeq™ DNA Signature Prep kit pre-PCR reagent chemistry. Assessment of the pH levels of the STR GO! Lysis buffer (QIAGEN) indicated a buffer of a highly alkaline nature (pH = 12).

### Phase 3: method optimisation

#### SwabSolution™ lysate library quality

**Fig. 1:**
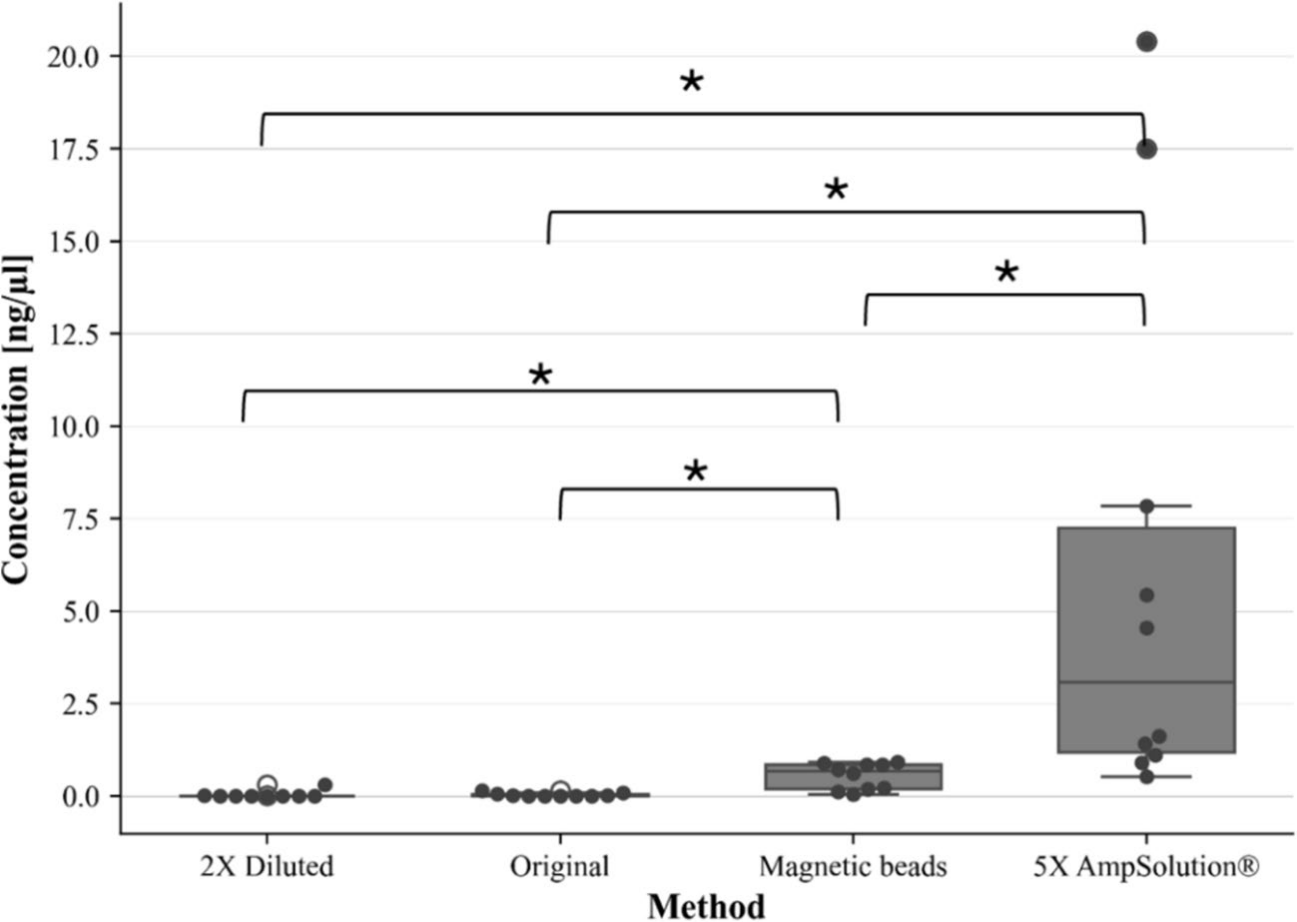
Box-and-whisker plot representing average library concentration for SwabSolution™ lysates prepared with modifications to the manufacturer’s protocol. Pairwise comparisons resulting in p-values < 0.05 are illustrated with a square bracket and asterisk (*)

Libraries prepared with 5X AmpSolution^®^ reagent resulted in the highest average library concentrations, followed by lysates prepared with magnetic bead purification and lysates prepared according to the manufacturer's protocol (i.e., “original”) (Fig. 1). Lysates diluted two-fold prior to library preparation resulted in the lowest library concentrations. Lysates spiked with 5X AmpSolution^®^ resulted in significantly higher library concentrations than all other optimisation methods tested (*p* < 0.05). Furthermore, lysates that underwent magnetic bead purification also resulted in significantly higher library concentrations than lysates prepared with the manufacturer's protocol and lysates diluted two-fold (*p* < 0.05). No significant differences were observed between library concentrations of lysates diluted two-fold and lysates prepared according to the manufacturer’s protocol.

**Fig. 2:**
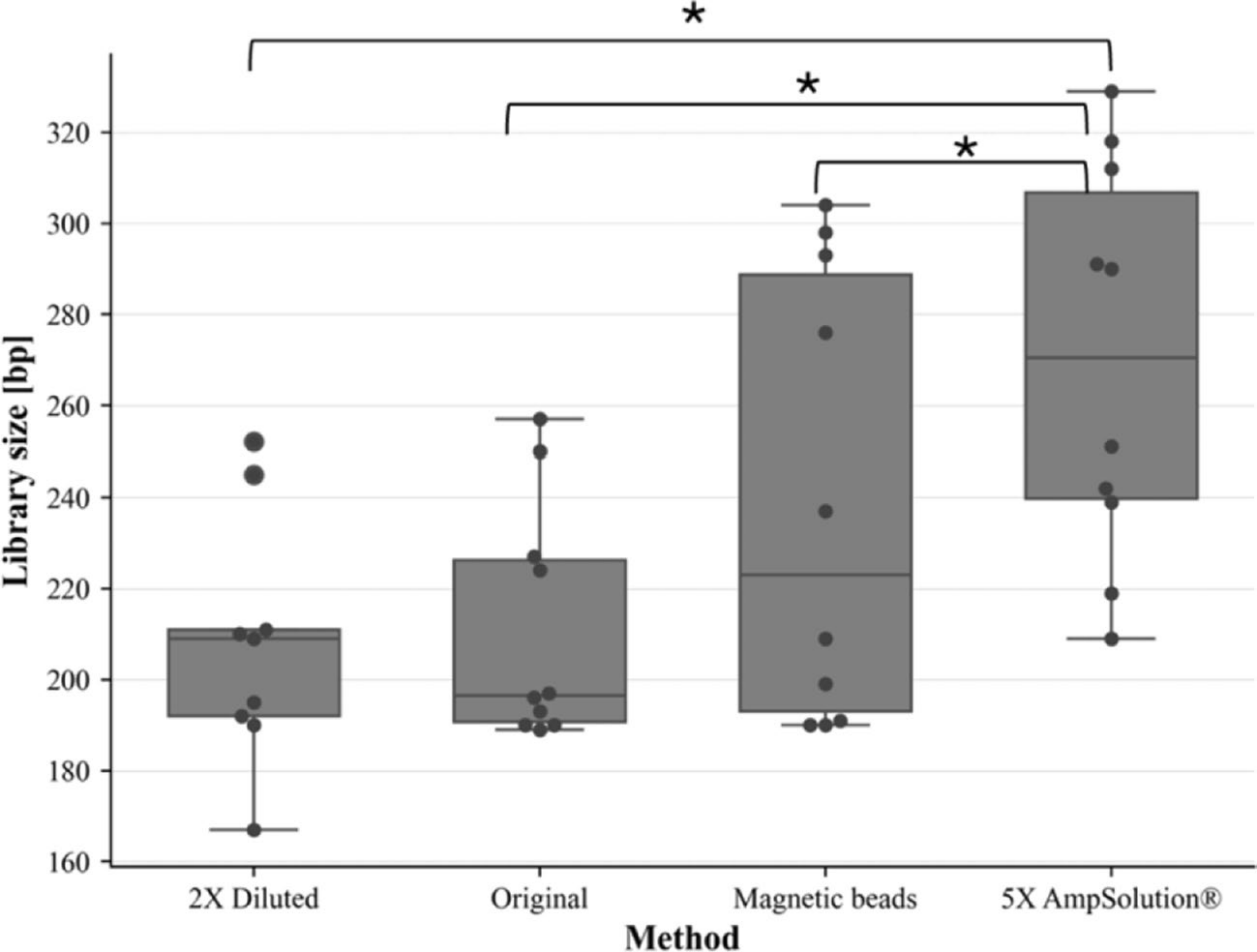
Box and whisker plot representing average library size (bp) for SwabSolution™ lysates prepared with modifications to the manufacturer’s protocol. Pairwise comparisons resulting in p-values < 0.05 are illustrated with a square bracket and asterisk (*)

**Fig. 3:**
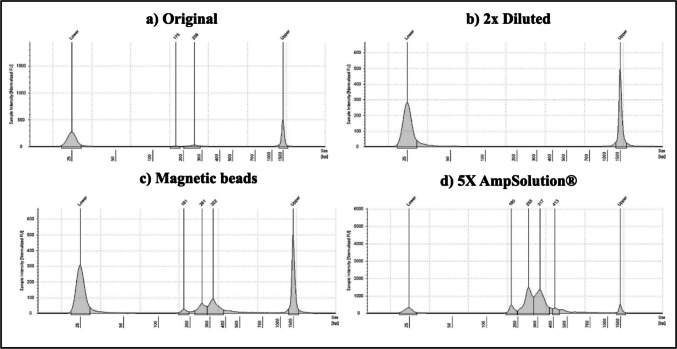
TapeStation traces with sample intensity shown on each Y-axis in fluorescence units and library size shown in base pairs (bp) on the X-axis. The traces are shown for a SwabSolution™ crude buccal swab lysate prepared with (a) the manufacturer’s protocol, (b) a modified protocol where the lysate was diluted 2x with nuclease-free water, (c) modified protocol whereby the lysate was purified using the Mag-Bind^®^ Blood DNA HV kit and (d) a modified protocol where 3 µL of 5X AmpSolution^®^ reagent was added to the PCR 1 reaction

Lysates prepared with 5X AmpSolution^®^ resulted in the highest overall library size compared to lysates purified with magnetic beads, lysates diluted two-fold, and lysates prepared with the manufacturer’s protocol (Figs. 2 and 3). Friedman’s test revealed significant differences between groups, while post-hoc comparisons revealed that significant differences in library size existed between lysates prepared with 5X AmpSolution^®^ reagent and all other methods tested (*p* < 0.05).

#### Sequencing success of optimised crude buccal swab lysate protocol: SwabSolution™ lysates

**Fig. 4:**
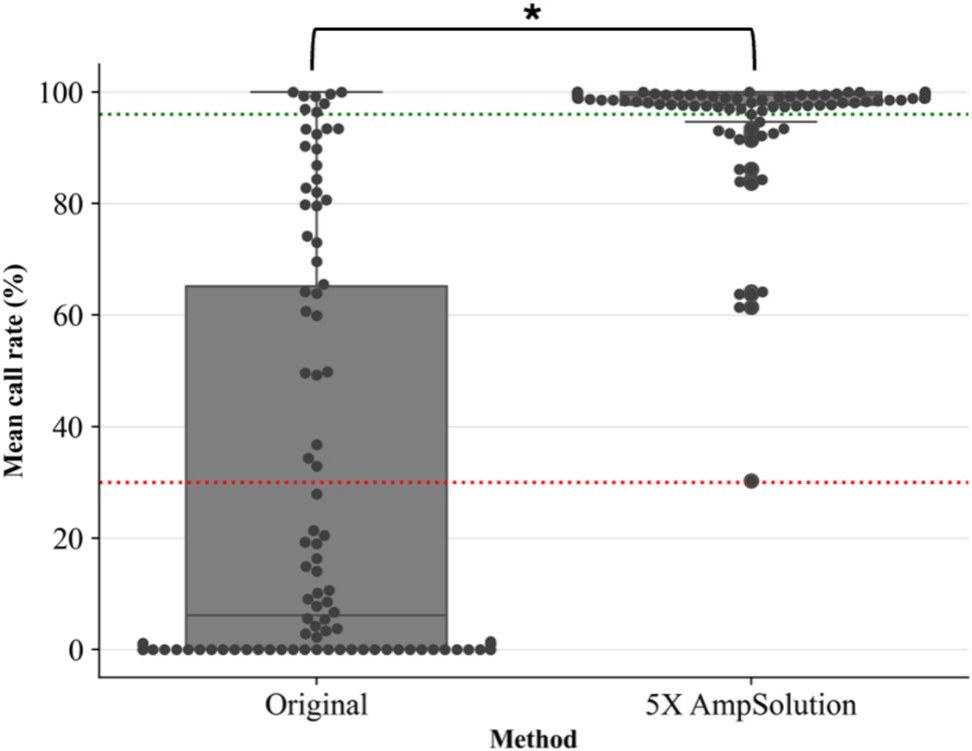
Box-and-whisker plot comparing call rates in percentage (%) across DPMA markers for SwabSolution™ lysates processed before (Original) and after optimisation (5X AmpSolution^®^ added). The red dotted line represents the mean call rate (30.03%) for lysates processed using the original protocol, while the green dotted line represents the mean call rate (96.39%) for lysates processed with 5X AmpSolution^®^. The asterisk (*) represents a p-value of < 0.05

The addition of 5X AmpSolution^®^ reagent improved both the quantity and quality of libraries generated for SwabSolution™ lysates. This method was selected to be used in the subsequent population study, as a direct PCR approach could be maintained. SwabSolution™ lysates that previously failed, as well as previously unprocessed SwabSolution™ lysates resulted in improved call rates (Fig. 4). The mean call rate for DPMA markers across 93 SwabSolution™ lysates processed before optimisation was 30.03 ± 37.72%. This increased to 96.39 ± 10.71% across 136 samples when adding 5X AmpSolution^®^, and this increase was statistically significant (*p* < 0.05). There were nine samples (*n* = 9) that resulted in partial profiles (call rates between 30.13% and 64.13%) even after 5X AmpSolution^®^ was added, but upon evaluation of qPCR data, these were found to be moderately degraded.

#### STR GO! Lysate library quality assessment

**Fig. 5:**
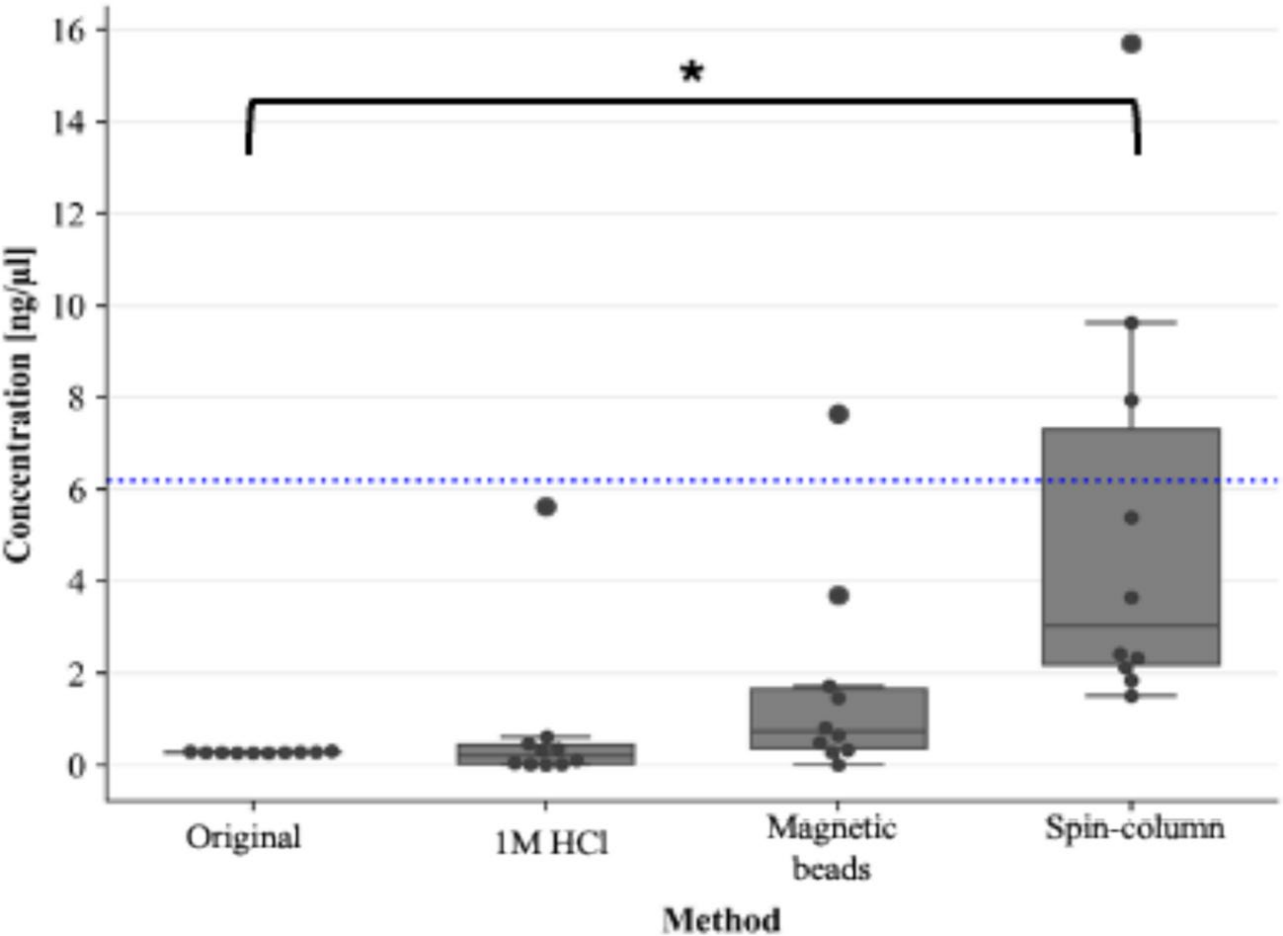
Box-and-whisker plot representing library concentration for STR GO! lysates prepared with modifications to the manufacturer’s protocol. The blue-dotted line represents the library concentration of the 2800 M control DNA library of 6.26 ng/µL. Pairwise comparisons resulting in p-values < 0.05 are illustrated with a square bracket and asterisk (*)

Lysates purified with the QIAamp^®^ DNA Investigator kit (QIAGEN) resulted in higher library concentrations than all other methods (Fig. 5). Significant differences in library concentration occurred between lysates purified with the QIAamp^®^ DNA Investigator kit and the lysates which were modified to include a 1 M HCl addition (*p* < 0.05) (Fig. 5). Although a marked improvement in library concentration was observed when using magnetic bead purification, the median concentration was lower than that observed for the 2800M control DNA (6.26 ng/µL).

**Fig. 6:**
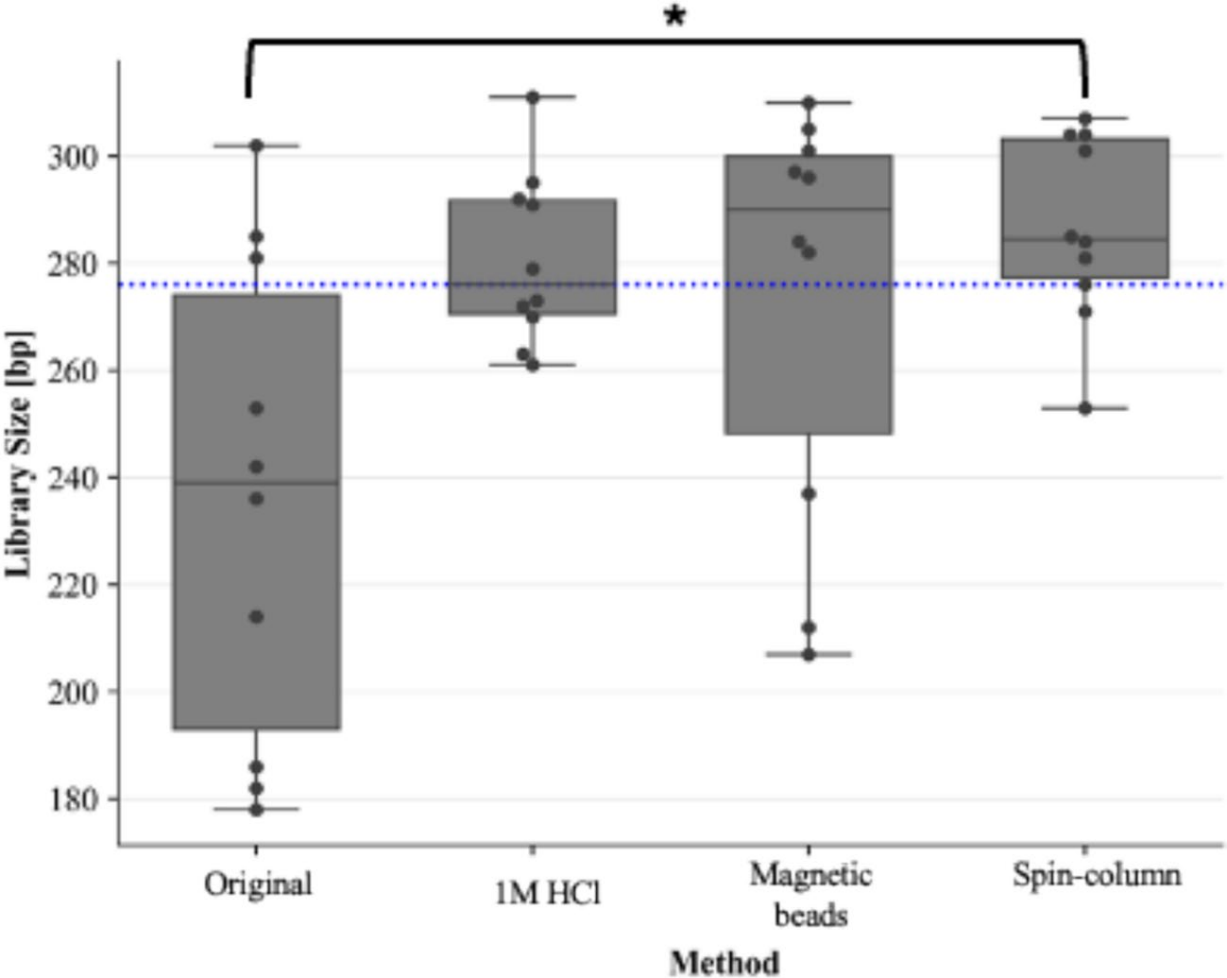
Box-and-whisker plots representing average library size for STR GO! lysates prepared with modifications to the manufacturer’s protocol. The blue-dotted line represents the average library size of the 2800 M control DNA library of 276 bp. Pairwise comparisons resulting in p-values < 0.05 are illustrated with a square bracket and asterisk (*)

For assessment of library size, lysates purified with the QIAamp^®^ DNA Investigator kit (QIAGEN) resulted in a significantly higher average library size (Median: 284.5 bp ± 17.49) than lysates prepared according to the manufacturer’s protocol, which resulted in the lowest library sizes when all methods were compared [(Median: 239 bp ± 45), (*p* < 0.05)] (Fig. 6).

**Fig. 7:**
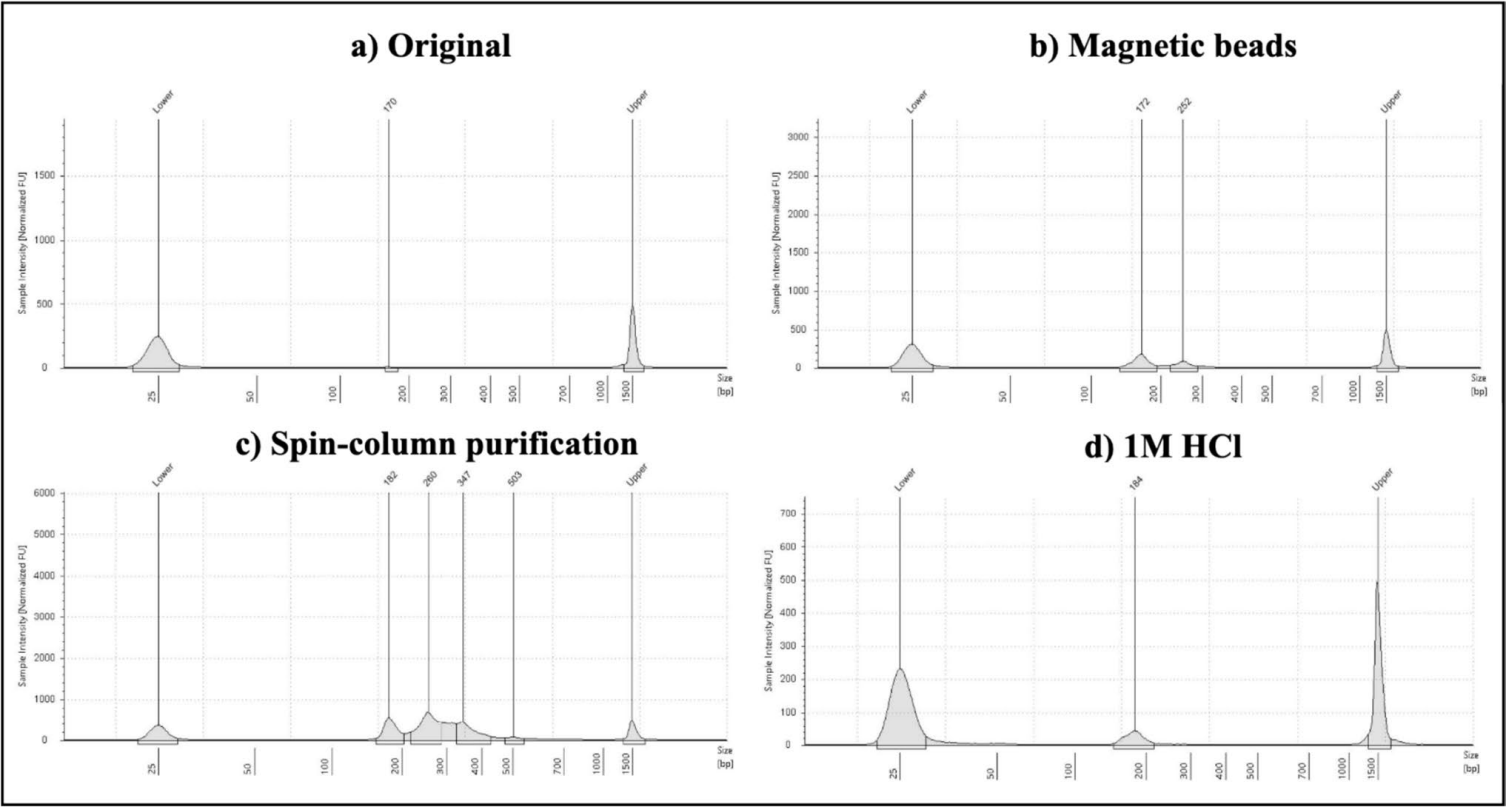
TapeStation traces with sample intensity shown on each Y-axis in fluorescence units and library size shown in base pairs (bp) on the X-axis. The traces are shown for a STR GO! crude buccal swab lysate prepared with (a) the manufacturer’s protocol, (b) a modified protocol whereby the lysate was purified using the Mag-Bind Blood DNA HV kit, (c) a modified protocol whereby the lysate was partially purified using spin-columns with the QIAamp^®^ DNA Investigator kit and (d) a modified direct-PCR protocol, whereby 1 M HCl was added directly to the PCR 1 reaction

As both purified lysates and the 2800 M control DNA had the same input amount (1 ng), they could be directly compared. A one-sample Wilcoxon test to compare library concentrations of each method of lysate preparation against the average library concentration of the control DNA indicated that the average library concentration of the 2800 M control DNA library resulted in significantly higher library concentrations than lysates prepared with; (a) 1 M HCl, (b) the manufacturer’s protocol (c) magnetic bead purification (*p* < 0.05). Only the *average library concentration* of lysates prepared with the QIAamp^®^ DNA Investigator kit did not differ significantly from the library concentration of the control DNA library (*p* > 0.05). There were no significant differences in *average library size* for all methods compared to the 2800 M control DNA library size of 276 bp (Figs. 6 and 7).

#### Sequencing success of optimised crude buccal swab lysate protocol: STR GO! Lysates

**Fig. 8:**
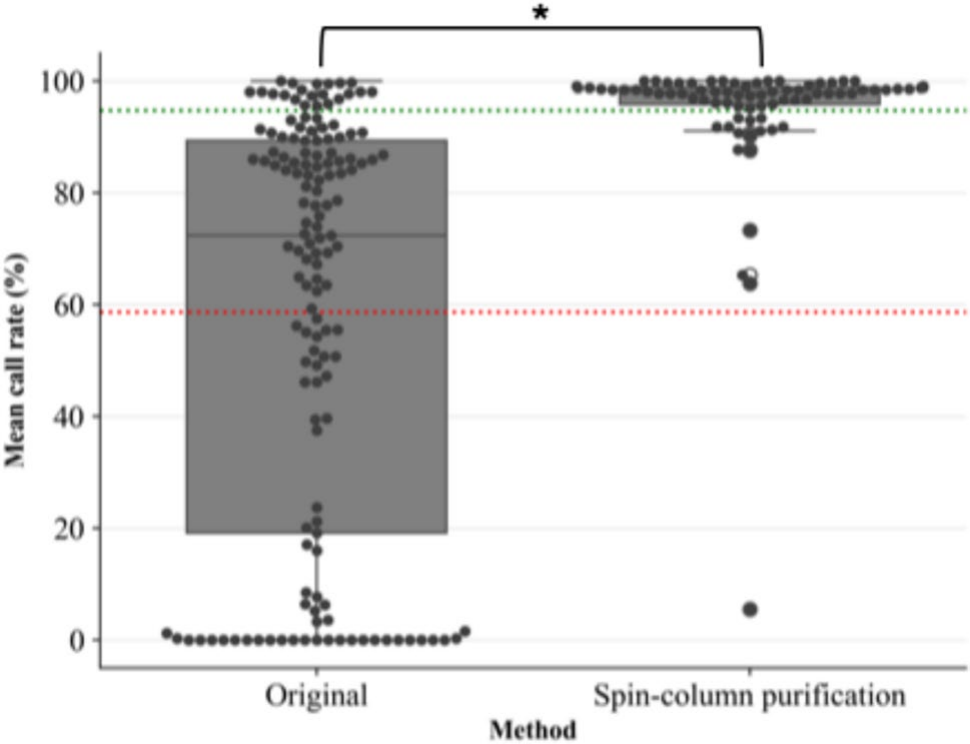
Box-and-whisker plot comparing call rates in percentage (%) across DPMA markers for STRGO! lysates processed before (Original) and after optimisation (Spin-column purification)). The red dotted line represents the mean call rate (58.64%) for lysates processed using the original protocol, while the green dotted line represents the mean call rate (93.38%) for lysates purified using spin-column purification. The asterisk (*) represents a p-value of < 0.05

It was observed from the results that purification with the QIAamp^®^ DNA Investigator kit resulted in the most improved library concentration. Although average library sizes were similar across all methods, the addition of 1 M HCl and silica-based purification resulted in a more consistent average library concentration across the 10 samples, showing the least variation. However, the addition of 1 M HCl resulted in extremely low concentrations when compared to the silica-based method. For this reason, STR GO! lysates that had failed in the initial testing phase, as well as previously unprocessed STR GO! lysates were purified using spin-column purification. A marked improvement in call rate was noted, where the original protocol resulted in a mean call rate of 58.64 ± 36.85% across 145 samples, increasing to 94.73 ± 12.27% when using the optimised protocol across 76 samples (Fig. 8). This increase was statistically significant (*p* < 0.05). Thus, for both SwabSolution™ and STR GO! lysates, the average call rate improved from 44.35% to 93.38%.

## Discussion

The aim of this study was to develop an optimised approach toward processing crude buccal swab SwabSolution™ and STR GO! lysates with the ForenSeq™ DNA Signature prep kit, given suboptimal first-time success rates with the manufacturer’s recommended protocol. The addition of 5X AmpSolution^®^ to SwabSolution™ lysates significantly improved library quantity and quality compared to other methods (dilution and purification) (Figs. 1 and 2). Purification of STR GO! lysates using spin column-based methods resulted in overall improved library quantity and quality. The mechanisms by which library quality and quantity have been improved through these modifications are highlighted in this discussion.

### PCR inhibition in SwabSolution™ lysates

It was hypothesised that lysates contained PCR inhibitors interfering with ForenSeq™ kit PCR buffers. For lysates prepared with SwabSolution™ (Promega), the PCR inhibition hypothesis was confirmed with real-time PCR, as SwabSolution™ lysates had IPC C_T_ values above 31, and when diluted two-fold, resulted in IPC C_T_ values below 31. This suggests that if a PCR inhibitor was present in the undiluted lysate, the effect of diluting the sample would essentially dilute the inhibitor, thereby enabling successful qPCR amplification.

Previous studies have shown high success rates with the SwabSolution™ kit (Promega) when applied to conventional DNA profiling methods [30–32]. For the samples used in this study, all SwabSolution™ lysates resulted in high success with CE kits [17, 18]. The ForenSeq™ DNA Signature Prep kit manufacturer’s protocol does not indicate that quantification of the lysate, or library quality assessment is required [19]. However, studies that have used the SwabSolution™ kit (Promega) with the ForenSeq™ DNA Signature Prep kit are limited. In one such study, authors stipulated that a direct PCR approach was not used, but rather that lysates were purified, quantified and diluted prior to processing with MPS kits [33].

In another study using the same kits, authors quantified and diluted lysates to 0.2 ng/µL prior to processing [34]. Therefore, neither of these studies employed a direct PCR approach when using the SwabSolution™ kit (Promega). Although it cannot be assumed that the authors also experienced first-time failure with the ForenSeq™ DNA Signature Prep kit when processing SwabSolution™ lysates, modifications were made to the manufacturer’s protocol, suggesting that authors suspected that using a full direct PCR approach would have a higher risk of sub-optimal first-time success rates with MPS [33, 34].

In this study, three methods were evaluated and compared to overcome PCR inhibition in SwabSolution™ lysates, including (1) dilution with water; (2) purification with magnetic beads and (3); addition of 5X AmpSolution^®^. The first method (i.e., dilution with water) was not a suitable method for overcoming inhibition in SwabSolution™ lysates. The poor library quality of lysates diluted with water was surprising as quantification results showed an acceptable concentration, DI value and IPC C_T_ value. Real-time PCR has been used as a method for assessing downstream profiling success in many studies, however, a few sources have highlighted its shortcomings when using real-time PCR results to inform to profiling success [35–37]. In this study, real-time PCR was not a suitable and consistent proxy for determination of MPS profiling success of diluted SwabSolution™ lysates. However, it was successfully used to detect PCR inhibition in undiluted lysates. To this end, the Quantifiler^®^ Trio kit was more tolerant to PCR inhibitors in the SwabSolution™ lysates than the ForenSeq™ DNA Signature Prep kit and thus minimally informative for predicted MPS profile success.

The second method (i.e., purification with magnetic beads) resulted in lower IPC C_T_ values, suggesting that PCR inhibition was successfully overcome. Additionally, improved library quality (i.e., library concentration and size) was achieved when compared to libraries where lysates were diluted with water, but this improvement was not significantly better than libraries of lysates that had resulted in failed MPS profiles.

The third method (i.e., the addition of 5X AmpSolution^®^) significantly improved library concentration and library size, while showing similar concentration and library size to that of the 2800 M positive control. The use of 5X AmpSolution^®^ is not stipulated in the manufacturer’s protocol but is recommended when performing direct amplification of SwabSolution™ lysates on specific PowerPlex^®^ systems [15, 19]. Although the composition of the 5X AmpSolution^®^ reagent is proprietary, it is known that the reagent works to reduce the effect of inhibitors in the lysate. This approach was selected as the best method to overcome inhibition, while maintaining a direct-PCR approach. It is therefore recommended that when processing SwabSolution™ lysates using a direct PCR approach with the ForenSeq™ DNA Signature Prep kit, 5X AmpSolution^®^ must be added to the reaction to maximise the first-time success rates. Furthermore, the manufacturers have developed an enhanced PCR 1 (ePCR 1) buffer designed to overcome inhibition in hard tissue samples, however, the manufacturer’s protocol states that this buffer should *not* be used when processing crude buccal swab lysates, but no information on why it should not be used is given. Although, the components in this buffer may play a role in improving amplification efficiency, further investigation is recommended to determine the influence of the ePCR 1 buffer on inhibitors present in crude lysates [19].

### Limited pH buffering capacity in STR GO! Lysates

Two purification methods (spin-column and magnetic beads) and a direct PCR approach (addition of 1 M HCl) was tested to optimise STR GO! lysates. While both purification methods resulted in improved library concentrations compared to the original method, the spin-column purification resulted in higher library concentrations than magnetic-bead based purification, and significantly higher concentrations than the direct approach. Although a slight improvement was noted with the Mag-Bind^®^ Blood DNA HV kit, recovery was lower than with the QIAamp^®^ DNA Investigator kit (QIAGEN). This is likely due to improved compatibility between the QIAamp^®^ DNA Investigator Kit (QIAGEN) and the STR GO! Lysis buffer (QIAGEN) components.

Although spin-column purification resulted in adequate library quality and concentration, the method steers away from using a direct PCR approach. A direct-PCR approach was tested on STR GO! lysates by the addition of an acid to the PCR reaction to reduce the pH of the lysate to an acceptable range. However, this was at the cost of diluting the lysate to a level that was found to be too low for efficient PCR amplification.

A highly alkaline environment is usually overcome through built-in buffering capacity of PCR reagents in CE kits [38]. It is also known that CE kits have been developed and optimised to tolerate high inhibitor concentrations. However, it is anticipated that the sensitivity of MPS kit chemistries may not include sufficient buffering capacity to reduce pH to an acceptable range for optimal polymerase activity [39]. For this reason, it is recommended that the manufacturer’s produce a reagent that would enable the direct amplification of samples lysed in STR GO! Lysis buffer (QIAGEN) that addresses the high pH, while simultaneously maintaining a suitable input concentration needed for successful amplification of fragments. This is especially important for laboratories already making use of STR GO! lysates for reference buccal swabs used for large-scale sequence-based population studies.

#### Importance of a quality control step prior to and post library preparation when conducting databasing studies

The low first-time success rates of lysates pose challenges for concordance tests in MPS population studies. This is particularly true for data generated using STR GO! lysates, as in Heathfield et al., 2024, and Whittaker and Heathfield, 2024, unless protocol modifications are made [17, 18].

Considering this, an update to the manufacturer’s protocol is proposed to purify STR GO! lysates prior to library preparation or to include a pre-sequencing quality control step on a small subset of purified libraries of STR GO! lysates. This study has demonstrated that TapeStation is a suitable quality control method to review the quality of library traces prior to normalisation and sequencing. Although not required in the manufacturer's protocol, the use of crude buccal swab lysates with the ForenSeq™ DNA Signature Prep kit does in fact require quality control steps prior to library preparation and prior to sequencing.

## Conclusion

Forensic laboratories moving towards the adoption of MPS for forensic casework or reference samples should consider the compatibility of previously collected samples used for direct PCR with CE technology with sensitive MPS kit chemistries. This paper has provided valuable insights into direct PCR approaches that can be adapted for MPS with the ForenSeq™ DNA Signature Prep kit. The systematic approach used to elucidate and identify optimal methods for sequencing of crude buccal swab lysates have informed recommendations for current and future researchers conducting large-scale MPS studies. For SwabSolution™ lysates, the addition of 5X AmpSolution^®^ to the lysate prior to PCR steps is recommended to overcome potential PCR inhibition, while spin-column purification of the STR GO! lysate is recommended. The insights herein have therefore achieved the aim of improving low first-pass success rates obtained with the SwabSolution™ and STR GO! lysates when processed using MPS. Although this study has only assessed the performance of crude lysates with the ForenSeq™ DNA Signature Prep kit alone, similar performances of these crude lysates with other ForenSeq™ kits (ForenSeq™ MainstAY, ForenSeq™ Kintelligence) may be obtained, although this requires testing prior to large-scale use. The results of this study have streamlined the manufacturer’s protocol to avoid repeat sampling and re-sequencing. This is crucial for low-resource laboratories that may rely on readily available population or reference samples for establishing allele frequency databases, avoiding significant financial constraints.
